# Knowledge of the health consequences of tobacco smoking: a cross-sectional survey of Vietnamese adults

**DOI:** 10.3402/gha.v6i0.18707

**Published:** 2013-01-31

**Authors:** Dao Thi Minh An, Hoang Van Minh, Le Thi Huong, Kim Bao Giang, Le Thi Thanh Xuan, Phan Thi Hai, Pham Quynh Nga, Jason Hsia

**Affiliations:** 1 Department of Epidemiology, Institute for Preventive Medicine and Public Health, Hanoi Medical University, Hanoi, Vietnam; 2 The Vietnam Steering Committee on Smoking and Health, Hanoi, Vietnam; 3 World Health Organization Office in Viet Nam, Hanoi, Vietnam; 4 Center for Disease Control and Prevention, Atlanta, USA

**Keywords:** knowledge, smoking, health consequences, global adult tobacco survey, Vietnam

## Abstract

**Background:**

Although substantial efforts have been made to curtail smoking in Vietnam, the 2010 Global Adult Tobacco Survey (GATS) revealed that the proportion of male adults currently smoking remains high at 47.4%.

**Objectives:**

To determine the level of, and characteristics associated with, knowledge of the health consequences of smoking among Vietnamese adults.

**Design:**

GATS 2010 was designed to survey a nationally representative sample of Vietnamese men and women aged 15 and older drawn from 11,142 households using a two-stage sampling design. Descriptive statistics were calculated and multivariate logistic regression was used to examine associations between postulated exposure factors (age, education, access to information, ethnic group etc.) and knowledge on health risks.

**Results:**

General knowledge on the health risks of active smoking (AS) and exposure to second hand smoke (SHS) was good (90% and 83%, respectively). However, knowledge on specific diseases related to tobacco smoking (stroke, heart attack, and lung cancer) appeared to be lower (51.5%). Non-smokers had a significantly higher likelihood of demonstrating better knowledge on health risks related to AS (OR 1.6) and SHS (OR 1.7) than smokers. Adults with secondary education, college education or above also had significantly higher levels knowledge of AS/SHS health risks than those with primary education (AS: ORs 1.6, 1.7, and 1.9, respectively, and SHS: ORs 2.4, 3.9, and 5.7 respectively). Increasing age was positively associated with knowledge of the health consequences of SHS, and access to information was significantly associated with knowledge of AS/SHS health risks (ORs 2.3 and 1.9 respectively). Otherwise, non-Kinh ethnic groups had significantly less knowledge on health risks of AS/SHS than Kinh ethnic groups.

**Conclusions:**

It may be necessary to target tobacco prevention programs to specific subgroups including current smokers, adults with low education, non-Kinh ethnics in order to increase their knowledge on health risks of smoking. Comprehensive messages and/or images about specific diseases related to AS/SHS should be conveyed using of different channels and modes specific to local cultures to increase knowledge on smoking health consequences for general population.

Over the past 10 years, Vietnam had made substantial efforts on tobacco control. In 2005, Vietnam ratified the Framework Convention on Tobacco Control (FCTC), and in August 2009, the Prime Minister's Decision No. 1315/QD-TTg approved a plan to implement FCTC. The Ministry of Health, Ministry of Education & Training, and Ministry of Transport also issued official directives for the implementation of a smoke-free policy. At the same time, levels of smoking remain high in Vietnam. In 2001–02, 69.1% of men smoked cigarettes; 23.2% of men smoked water pipe tobacco. In addition, 63% of households had at least one smoker, and 71% of children under age 5 lived in households with at least one smoker (1). In 2003, nearly 60% of school-age youth reported being exposed to secondhand smoke at home (2). The prevalence of cigarette smoking among students was 3.3% overall, while that among male students was 5.9% (3). The 2010 Global Adult Tobacco Survey (GATS) in Vietnam showed that smoking prevalence among adult males was 47.4%, and in a survey conducted in hospitals and public places evidence revealed that smoking was common (4, 5).

**Policy Recommendations**

- IEC activities in Vietnam should be designed specifically to focus on specific target populations, given their smoking status and demographic characteristics.
- Information conveyed to smokers should be comprehensive, including not only general messages of smokinghealth harms but also specific health risks of active, secondhand smoking, and disease burdens of smoking based on the real data of specific diseases related to smoking which Vietnamese people suffered from.
- To improve accessibility to information of health consequences of smoking, messages that smoking is harmful should be conveyed appropriately, frequently, and efficiently to different targeted populations by different channels/modes of communication which are mostly preferred or mostly accessible by each targeted population in the Vietnamese setting.

Knowledge is defined by the Oxford English Dictionary as expertise and skills acquired by a person through experience or education; while perception is the process by which humans interpret and organize sensation to produce a meaningful experience of the world (6). It is now well established that if people's perceptions of the commonality and acceptability of a behavior can be adjusted, their inclination to engage in that behavior may be influenced. For example, the more common and acceptable young people think smoking is among their immediate peers, their family group and society as a whole, the more likely they are to take up the habit (7). Conversely, smoking uptake can potentially be reduced if pro-smoking norms are challenged and anti-smoking norms are strengthened. Gerards Hastings in his book titled ‘Social Marketing – Why should the devil have all the best’ wrote that ‘Normative education or de-normalization programs, therefore, can correct erroneous perceptions of the prevalence and acceptability of drug and alcohol use and establish conservative group norms that are postulated to operate through lowering expectations about prevalence and acceptability of use and the reduced availability of substances in peer-oriented social settings’.

Therefore, Article 4 of FCTC of the World Health Organization (WHO) stated that every person should be informed of the health consequences, addictive nature, and mortal threat posed by tobacco consumption and exposure to tobacco smoke. As a result, several countries have attempted to educate their populations about the health consequences of smoking (8, 9). The Vietnam Steering Committee on Smoking and Health (VINACOSH) has made significant efforts to convey messages of tobacco control to Vietnamese population during the past 10 years by several Information, Education, and Communication (IEC) activities in cooperation with the International Development Establishment (IDE). These activities consisted of celebrity television interviews; tobacco control communication activities in combination with a trans – Viet bicycle riding tour from Hanoi to Ho Chi Minh City; hosting training IEC workshops on designing a smokers’ behavior influencing project, called ‘Keep tobacco smoke away from your wife and kids’ in Hai Phong City; and improving public awareness on the negative effects of tobacco on human health and the economy. VINACOSH has been cooperating with IDE in developing tobacco and secondhand smoke control IEC materials, applying social marketing approaches in designing IEC materials, and selecting relevant communication channels (10). Although many efforts have been made under the IEC programs in Vietnam, there is still a need for additional research to understand the relationship of knowledge of smoking-health risks and smoking behavior in Vietnam. The purpose of this study was to identify the level of knowledge of health consequences of tobacco smoking and its associated factors to inform IEC programs on reducing tobacco smoking.

## Methods

### Selection and description of participants

Data used in this article were obtained from the 2010 GATS Vietnam, which was part of the multi-countries household survey of the ongoing Global Tobacco Surveillance System (GTSS) (11). The GATS in Vietnam was designed as a nationally representative survey of all non-institutionalized men and women aged 15 and older, with their primary residence in Vietnam.

A total of 11,142 households were selected using a two-phase sampling design analogous to a three-stage stratified cluster sampling. In 2009, the General Statistics Office (GSO) in Vietnam conducted a population and housing census. Meanwhile, GSO prepared a 15% master sample to serve as a future national survey-sampling frame. The 15% master sample contains a subset of enumeration areas (EAs) that consist of 15% of the population in Vietnam stratified into three groups. The first group consists of 132 districts, towns, or cities of provinces. The second group consists of 294 plain and coastal districts. The third group consists of 256 mountainous and island districts. The GATS sample was drawn from the 15% master sample after further stratification of the three groups into urban and rural areas (six strata in total).

At the first stage of sampling, the primary sampling unit (PSU) was EAs. The sampling frame was a list of the EAs, from the 15% master sample, with the number of households as well as identifiable information, administered by the GSO Vietnam in 2009 from the census. For each of the six strata, the designated number of EAs was selected. A selection probability proportional to size (PPS) sampling method was used, where the size was the selection probability of an EA using PPS sampling from the entire target population divided by the selection probability of an EA for the master sample.

At the second stage of sampling, 18 households from the selected urban EAs and 16 households from the selected rural EAs were chosen using simple systematic random sampling. One eligible household member from each selected household was then randomly chosen for an interview.

Note that the current design and the design where EAs were sampled directly universally were analogous. The selection probability of an eligible individual was calculated as a product of selection probability for each stage. The sampling base weight for an eligible individual was the inverse of the selection probability shown above.

### Data collection

Handheld computers (iPAQ) were used for collecting data. Interviewers and supervisors from GSO conducted fieldwork, under the co-supervision of the WHO in Vietnam and Hanoi Medical University. The fieldwork lasted from March 22, 2010, to May 13, 2010, in all 63 provinces of Vietnam. The interviewers and supervisors were experienced, trained in using computers and handheld (iPAQ) devices, and had previous experience working with local authorities, which is key to minimizing non-response rates. A case file containing addresses and names of the households assigned to the interviewer was preloaded in the iPAQ prior to the fieldwork. The data collectors went to the residences of the respondents and met the head of the household to acquire general information about the number of eligible individuals in the household. This number was entered into the handheld computer and one individual was automatically selected to be interviewed. All responses were entered in the iPAQ.

### Variables and definition used in this article

#### Dependent variables

1. Knowledge of specific diseases of active smoking defined by one who answered ‘yes’ to all four situations (smoking causes serious illness, stroke, heart attack, and lung cancer)
2. Knowledge of health consequences of secondhand smoke defined by one answered ‘yes’ to question ‘breathing other people's smoke cause serious illness in non-smokers’.

#### Independent variables

1. Smoking status: current smokers and current non-smokers
2. Demographic variables: age; sex; education, quintile of household income which is based on the current study; area (urban/rural); region (ecological area), ethnic group
3. Access to positive information channel in the last 30 days (Answer ‘yes’ to one of the following: information about health consequences of smoking, encouragement to quit, or health warnings on cigarette packages)
4. Access to negative information channel in the last 30 days (Answer ‘yes’ to one of the following: cigarette advertisement through media, cigarette advertisement events, or cigarette promotion).

### Statistics

Descriptive and statistical analyses with percentages and 95% confidence interval (CI) were calculated using Stata 10 software (Stata Corporation). The relationship between demographic variables (sex, areas, education, age group, quintiles of income, regions), smoking status, and levels of access to information and knowledge of health consequences were conducted. Multivariate logistic regression modeling was performed to identify what variables associated with knowledge of health consequences of active smoking and passive smoking in which variables of demographic characteristics, smoking status, rule of ‘no smoking at home’ and levels of access to information were screened for bivariate association and then all entered into the model as the independent factors. Backward elimination was used to remove ones that were not statistically significant (*p>*0.05). The odds ratio (OR) with 95% CI was used. Sampling weights were used in all of the computations.

## Results

The household response rate was 96.9% and was a little higher in rural areas than that in urban areas (97.5 and 96.5%, respectively). The individual response rate was 95.7% and was also a little higher in rural areas than that in urban areas (96.3 and 95.0%, respectively). Overall, 0.6% of the households and 0.6% of the selected individuals refused to respond to the survey. The total response rate was 92.7% (93.9% in rural areas and 91.7% in urban sites).

Among 9,925 completed interviews of adults aged 15 and above, two-thirds were living in rural areas. People aged 25–44 made up the largest proportion (41.9%). The educational level that predominated was secondary school (52.5%), followed by primary or less (26%), while college degree or above was only 7.2% of the total. The main occupation of the study population was farmers (49.6%), followed by service/sales (19.2%) and production/driving (12.9%). By ethnicity, 84.5% were Kinh people, and the remaining 15.5% belonged to other ethnic groups. By marital status, 67.7% of the total was married, 26.2% were single, and the remaining 6.2% were separated/divorced/widowed (Table 1).

**Table 1:** Distribution of adults ≥15 years by selected demographic characteristics – Viet Nam GATS, 2010

	Weighted		
		
Demographic characteristics	Percentage (95% CI*)	Number of adults (in thousands)	Unweighted number of adults
Overall	100	64,321	9,925
Gender			
Male	48.6 (47.3–49.9)	31,259	4,356
Female	51.4 (50.1–52.7)	33,063	5,569
			
Age (years)			
15–24	25.9 (24.6–27.2)	16,637	1,656
25–44	41.9 (40.6–43.2)	26,944	4,251
45–64	23.4 (22.4–24.5)	15,065	2,886
65+	8.8 (8.2–9.5)	5,675	1,132
			
Residence			
Urban	30.7 (30.0–31.4)	19,725	4,958
Rural	69.3 (68.6–70.0)	44,596	4,967
			
Education level			
Primary or less	26.0 (24.2–27.8)	12,377	2,034
Lower secondary	52.5 (50.8–54.3)	25,031	3,981
Upper secondary	14.3 (13.1–15.5)	6,794	1,023
College or above	7.2 (6.6–7.9)	3,447	1,227

* 95% confidence interval.

N=9,925 individuals from 656 PSUs of 6 strata.

Generally, the percentage of adults who agreed that active smoking and exposure to secondhand smoke causes serious illness were at high levels (93.4 and 83.8%, respectively) but differed by demographic characteristics. Regarding knowledge of harmful health effects of active and passive smoking, adults living in urban areas were more knowledgeable than those living in rural areas (97.1 vs 92.1% and 90.8 vs 81.3%, respectively); Kinh ethnic had greater knowledge than non-Kinh ethnic (96.8 vs 84.3% and 88.2 vs 72.0%, respectively). The respondents who had higher income and education were more likely to have better knowledge than those who had not. There were no differences for knowledge of health damage by sex, age group, and ecological region (Table 2).

**Table 2:** Knowledge of health consequences of tobacco smoking by demographic characteristics

	Active smoking causes serious illness	Secondhand smoking causes serious illness
	
Demographic characteristics	Percentage (95% CI*)	Percentage (95% CI*)
Over all	93.4 (91.0–95.2)	83.8 (81.3–86.1)
Sex		
Male	93.7 (91.9–95.1)	84.3 (81.8–86.4)
Female	93.2 (89.9–95.4)	83.4 (80.3–86.1)
		
Residence		
Urban	97.1 (96.4–97.6)	90.8 (89.7–91.8)
Rural	92.1 (88.8–94.5)	81.3 (78.0–84.2)
		
Ethnic group		
Kinh	96.8 (96.1–97.3)	88.2 (87.0–89.2)
Other	84.3 (76.6–89.8)	72.0 (64.7–78.2)
		
Ecological regions		
Red River Delta	97.4 (96.6–98.0)	91.5 (90.2–92.7)
Northern midland and mountain	96.1 (94.6–97.2)	86.2 (82.9–88.9)
North Central area and Central coastal	97.1 (95.0–98.3)	92.6 (90.0–94.5)
Central highlands	98.0 (94.5–99.3)	93.0 (84.6–97.0)
South East	95.1 (93.8–96.2)	82.5 (80.0–84.8)
Mekong River Delta	88.6 (82.1–92.9)	78.9 (72.5–84.1)
		
Age groups		
15–24	93.9 (90.6–96.0)	89.7 (86.1–92.4)
25–34	93.7 (89.0–96.5)	85.0 (79.6–89.1)
35–44	94.0 (89.3–96.7)	84.6 (81.0–87.6)
45–54	95.6 (94.1–96.7)	87.0 (84.8–88.9)
55–64	93.5 (90.1–95.8)	79.1 (75.1–82.6)
>64	87.1 (83.7–89.9)	70.0 (66.1–73.6)
		
Incomes		
Quintile 1	85.3 (78.8–90.1)	69.6 (64.1–74.6)
Quintile 2	96.0 (94.5–97.1)	86.7 (84.1–89.0)
Quintile 3	96.9 (95.6–97.8)	88.9 (86.5–90.8)
Quintile 4	97.9 (97.0–98.6)	92.1 (90.6–93.4)
Quintile 5	97.7 (96.8–98.3)	94.8 (93.5–95.8)
		
Education		
Primary or less	84.5 (79.0–88.7)	64.6 (60.3–68.7)
Lower secondary	96.8 (95.4–97.8)	88.7 (86.6–90.5)
Upper secondary	98.2 (96.9–99.0)	95.0 (93.1–96.3)
College and/or above	99.1 (98.4–99.5)	97.1 (95.8–97.9)

* 95% confidence interval.

N=9,919 individuals from 656 PSUs of 6 strata.

However, only 51.5% of interviewees answered correctly to all three specific health consequences (stroke, heart attack, and lung cancer). The most common health consequence was lung cancer (95.8%), while strokes and heart attacks were found to be much lower (67.6 and 60.9%, respectively). Of interest, current smokers displayed significantly lower knowledge of health risks of active smoking than current non-smokers, for example, smoking causes serious illness (83.3 vs 95.1%), stroke (59.4 vs 70.3%), heart attack (54.2 vs. 63.1%), lung cancer (93.0 vs 96.7%), all three main consequences (43.1 vs 54.3%), and secondhand smoke (77.3 vs 86.0%) (Table 3).

**Table 3:** Knowledge of health consequences of tobacco smoking by smoking status

Knowledge of health consequences of tobacco smoking	Current smokers	Current non-smokers	Total
Smoking causes	Percentage (95% CI*)	Percentage (95% CI*)	Percentage (95% CI*)
Serious illness	88.3 (82.8–92.2)	95.1 (93.6–96.3)	93.4 (91.0–95.2)
Stroke	59.4 (54.0–64.6)	70.3 (67.8–72.7)	67.6 (64.9–70.3)
Heart attack	54.2 (49.9–58.5)	63.1 (60.8–65.4)	60.9 (58.5–63.4)
Lung cancer	93.0 (89.9–95.2)	96.7 (95.8–97.3)	95.8 (94.6–96.7)
Stroke, heart attack, and lung cancer	43.1 (38.0–48.4)	54.3 (52.0–56.6)	51.5 (48.8–54.2)
Breathing other people's smoke cause serious illness in non-smokers	77.3 (71.3–82.4)	86.0 (84.2–87.7)	83.8 (81.3–86.1)

* 95% confidence interval.

N=9,919 individuals from 656 PSUs of 6 strata.

There were significant differences in the knowledge of health consequences for those who have access to positive information and those who did not, with those having access to information having more knowledge of health consequences of active smoking than those who did not, for example, knowledge of: serious illness 96.2 vs 76.3%, stroke 71.8 vs 41.1%, heart attack 64.5 vs 37.9%, lung cancer 97.7 vs 83.4%, and all three main health consequences 55.5 vs 27.0%. This relationship held for individuals having access to information about secondhand smoke exposure; 87.9% of individuals with access knew that breathing other people's smoke can cause serious illness in non-smokers, while among adults without access only 59.0% knew about the consequences. However, as demonstrated in Table 4, there were not many other differences between the groups.

**Table 4:** Knowledge of health consequences of tobacco smoking by different channels of accessing to information

	Access to positive information**		Access to negative information***			
		
Knowledge of health consequences of tobacco smoking	Percentage (95% CI*)		Percentage (95% CI*)	
Smoking causes	No	Yes	No	Yes
Serious illness	76.3 (67.5–83.3)	96.2 (94.9–97.2)	93.2 (90.9–94.9)	94.7 (90.1–97.2)
Stroke	41.1 (34.5–48.0)	71.8 (69.7–73.7)	66.5 (63.5–69.3)	75.1 (70.0–79.7)
Heart attack	37.9 (32.0–44.3)	64.5 (62.5–66.4)	59.6 (56.9–62.2)	69.6 (64.7–74.0)
Lung cancer	83.4 (77.7–87.9)	97.7 (97.1–98.1)	95.4 (94.1–96.5)	97.8 (96.6–98.6)
Stroke, heart attack, and lung cancer	27.0 (21.9–32.7)	55.5 (53.4–57.6)	50.1 (47.3–52.9)	60.0 (55.1–64.7)
Breathing other people's smoke cause serious illness in non-smokers	59.0 (52.0–65.6)	87.9 (86.0–89.6)	83.4 (80.9–85.6)	86.6 (82.2–90.0)

* 95% confidence interval.

** Access to positive information channel in the last 30 days (information about health consequences of smoking or encouragement to quit; health warnings on cigarette packages).

*** Access to negative information channel in the last 30 days (cigarette advertisement through media, cigarette advertisement events, cigarette promotion).

N=9,919 individuals from 656 PSUs of 6 strata.

Education level was reported only among respondents aged 25 years with the assumption that at age of 25 and above, people have completed their education and have acquired knowledge and attitudes about tobacco use. Two models were constructed: (1) Model a: for all of the study subjects (all aged 15 years and above) education was excluded and (2) Model b: for those aged 25 years and above and education was included as an independent variable. Model a had similar results as model b. In this article, model b was presented in Table 5 of the results section while model a was presented in Table 6 in the appendix section. Multivariate logistic regression analysis indicated that after adjusting for demographic characteristics, accessibility to information, rule of ‘no smoking at home’, and smoking status, predictors of knowledge of health consequences of active smoking are education, ethnicity, access to information, and smoking status. Adults at lower secondary, upper secondary, and college or above were more likely to have significantly better knowledge of health consequences of active smoking than those at primary school (OR: 1.6, 1.7, and 1.9, respectively). It was also the case of knowledge on health consequences of secondhand smoke (OR: 2.4, 3.9, and 5.7, respectively). Adults belonging to non-Kinh ethnic had significantly lower knowledge of active and passive smoking-health risks than Kinh ethnic (OR: 0.7 and 0.4, respectively). This model also indicated that accessing positive information had significant association with knowledge of both active and passive smoking-health risks (OR: 2.3 and 1.9, respectively). Noticeably, current non-smokers have significantly better knowledge of active and passive smoking-health risks than current smokers (OR: 1.6 and 1.7, respectively). Increasing age was positively related to knowledge of the health consequences of secondhand smoke (Table 5).

**Table 5:** Logistic regression analysis for factors associated with knowledge of health consequences of smoking (model b)

		Dependent variables			
		
		Knowledge on health risks of active smoking		Knowledge of health risks of secondhand smoking	
			
Independent variables	Sub-categories	OR**	95% CI*	OR**	95% CI*
Gender	Male	1			
	Female	0.9	0.8–1.1	1	0.7–1.4
					
Age group	25–34	1			
	35–44	0.9	0.7–1.2	2	1.5–2.7
	45–54	1	0.9–1.3	1.9	1.5–2.4
	55–64	1.2	1–1.5	2.2	1.6–2.9
	65 and above	1.3	1–1.6	1.3	1–1.7
					
Education	Primary	1			
	Lower secondary	1.6	1.3–1.9	2.4	1.9–3
	Upper secondary	1.7	1.3–2.2	3.9	2.6–5.8
	College or above	1.9	1.4–2.5	5.7	3.7–8.6
					
Income	Quintile 5	1			
	Quintile 1	0.9	0.7–1.1	0.5	0.3–0.6
	Quintile 2	0.9	0.7–1.1	0.8	0.6–1.1
	Quintile 3	0.8	0.7–1	0.7	0.5–1
	Quintile 4	0.9	0.8–1.1	0.9	0.6–1.2
					
Ethnic	Kinh	1			
	Non-Kinh ethnic	0.7	0.5–0.8	0.4	0.3–0.6
					
Access to positive information	No	1			
	Yes	2.3	2–2.6	1.9	1.6–2.3
					
Access to negative information	No	1			
	Yes	0.7	0.4–1.1	0.7	0.2–1.9
					
Area	Urban	1			
	Rural	1.1	1–1.3	1.1	0.9–1.3
					
Region	Red River Delta	1			
	Northern midland and mountain	1.2	0.9–1.5	1.9	1.2–3
	North Central area and Central coastal	1.2	1–1.4	1.1	0.8–1.5
	Central highlands	1.2	0.8–1.7	1.2	0.7–2.1
	South East	1.1	0.9–1.4	0.8	0.6–1.1
	Mekong River Delta	0.9	0.7–1.1	0.5	0.4–0.7
					
Smoking status	Current smokers	1			
	Current non-smoker	1.6	1.3–1.9	1.7	1.1–2.5

* 95% confidence interval

** odds ratio.

N=8,265 individuals from 656 PSUs of 6 strata.

## Discussion

This study found that although there was a high proportion among adults answering that active and secondhand smoking can cause serious illness (Table 1), only 51.5% of them understood that smoking can cause all three specific diseases (stroke, heart attack, and lung cancer) which were scientifically documented to have close relationships with smoking (Table 2) (12, 13). The finding that the risk of lung cancer was most frequently reported is consistent with other findings about the causes of disease reported by adults, even though heart disease is the number one killer of smokers (14).

The difference in knowledge between current smokers and current non-smokers was also studied elsewhere. Yang et al. found in the 2010 GATS China that current smokers were aware of fewer health effects of smoking than current non-smokers, respectively. For individual health effects, only 68% of current smokers agreed that smoking causes lung cancer in smokers while among current non-smokers, the percentage is more than 90%. In addition, only 36% of current smokers agreed that smoking causes coronary heart diseases while among current non-smokers the percentage is over 50% (15). The difference in knowledge of health risk between smokers and non-smokers is similar to patterns observed in China and Western countries, where smokers systematically underestimated their personal risks from smoking, presumably in an attempt to minimize cognitive dissonance from smoking and shield themselves from worry (16–18). Regarding differences in knowledge between Kinh ethnic and non-Kinh ethnic, the 2002 Vietnamese national health survey indicated that non-Kinh ethnic groups are people living in rural areas with lower levels of education than those living in urban areas (19), and a World Bank survey indicated that 90% of the poor in Vietnam live in the rural areas (20). ‘This has resulted in significant educational challenges’, as said by the Vice General Director of the Department of Sports, Entertainment and Economic Information at Viet Nam Television (VTV). In addition, in a very recent household survey in Vietnam conducted in 2011, it indicated that people at low education levels were more likely to smoke (21). This is where the IEC can play an important role in preventing smoking. Chee Ruey Hsieh in his study on knowledge of health risks in anti-smoking campaigns found that anti-smoking campaigns had a significantly positive effect on the public health-related knowledge (22). The Centers for Disease Control (CDC)'s best practice guidelines suggested that public education is an integral part in the efforts to both prevent initiation of tobacco use and to encourage tobacco cessation (23). This current study supports the importance of the ability to access information in both descriptive and multivariate analyses. Those accessing information of the health harms of active and secondhand smoking were 2.3 and 1.9 times, respectively, more likely to have more knowledge than those who did not. Returning to the first major model for communication in 1949 by Claude Elwood Shannon and Warren Weaver, the process of communication was broken down into eight discrete components, that is, information source, message, transmitter, signal, channel, noise, receiver, and destination. The current study has identified three out of eight components of this model that should be carefully considered when developing and carrying out an IEC program for tobacco control conducted in Vietnam.

First, by indicating that an understanding about specific health risks related to tobacco smoking among Vietnamese adults, especially among current smokers and non-Kinh ethnic, may still not be specific, this study can help inform IEC programs designed to prevent tobacco smoking; messages should be designed to be scientifically credible, comprehensive, and consistent for the nation as a whole. Second, by indicating that current smokers and non-Kinh ethnic groups have lower levels of knowledge than other groups, it may be necessary to target messages to individual population subgroups. Third, by indicating that access to positive information is predictive of knowledge, this current study highlights the importance of coverage of an IEC program. This issue is specially concerned in Vietnamese context because the GATS 2010 indicated that, percentage of population accessing to mass media was still very low (32.8%) and that accessing to health warnings on cigarette packets among current smokers was only 14% and among general population was only 12.7% (24).

Therefore, in terms of policy implication, it is necessary to develop a national IEC program for preventing smoking tobacco which would be designed for different target groups of adults, including a general one, smokers, and non-Kinh ethnic groups in which clear and comprehensive messages/images about the health harm of tobacco smoking is conveyed appropriately and efficiently by different channels/modes to local-specific cultures.

## Conclusion

The 2010 GATS in Vietnam showed that adults’ knowledge of specific diseases related to tobacco smoking was still vague as reflected in only 51.5% adults knowing that smoking can cause all three diseases of stroke, heart attack, and lung cancer. Regarding knowledge of health harms of active and passive smoking, current non-smokers were 1.6–1.7 times likely to have better knowledge than current smokers, respectively; non-Kinh ethnic groups were less likely to have knowledge (OR=0.7 and 0.4, respectively) than Kinh ethnic group smokers. Accessing positive information had a close association with knowledge of smoking-health risks (OR=2.3 and 1.9, respectively, with *p<*0.001). The more education adults had, the better knowledge of health consequences of tobacco smoking they got. Increasing age was positively related to knowledge of the health consequences of secondhand smoke.

## Appendix

**Table 6:** Logistic regression: factors associated with knowledge of health consequences of smoking (model a)

		Dependent variables			
		
		Knowledge on health risks of active smoking		Knowledge of health risks of secondhand smoking	
			
Independent variables	Sub-categories	OR**	95% CI*	OR**	95% CI*
Gender	Male	1			
	Female	0.8	0.7–1	0.8	0.5–1.1
					
Age group	25–34	1			
	35–44	0.8	0.6–1	3	2.2–4.1
	45–54	0.8	0.7–1.1	2.6	2–3.4
	55–64	1	0.8–1.2	3.1	2.3–4.1
	65 and above	0.7	0.5–0.9	1.7	1.2–2.2
					
Education	Primary	1			
	Lower secondary	1.6	1.3–1.9	2.4	1.9–3
	Upper secondary	1.7	1.3–2.2	3.9	2.6–5.8
	College or above	1.9	1.4–2.5	5.7	3.7–8.6
					
Income	Quintile 5	1			
	Quintile 1	0.7	0.6–0.9	0.3	0.2–0.4
	Quintile 2	0.8	0.6–1	0.5	0.4–0.7
	Quintile 3	0.8	0.6–0.9	0.5	0.4–0.7
	Quintile 4	0.9	0.7–1	0.7	0.5–1
					
Ethnic	Kinh	1			
	Non-Kinh ethnic	0.6	0.5–0.7	0.3	0.3–0.5
					
Access to positive information	No	1			
	Yes	2.4	2.1–2.8	2.2	1.8–2.6
					
Access to negative information	No	1			
	Yes	0.6	0.4–1.1	0.6	0.2–1.8
					
Area	Urban	1			
	Rural	1.1	0.9–1.2	1	0.8–1.2
					
Region	Red River Delta	1			
	Northern midland and mountain	1.2	0.9–1.5	1.8	1.1–2.9
	North Central area and Central coastal	1.2	1–1.4	1.1	0.8–1.5
	Central highlands	1.1	0.7–1.6	1	0.6–1.8
	South East	1	0.8–1.3	0.6	0.5–0.9
	Mekong River Delta	0.8	0.6–1	0.4	0.3–0.6
					
Regulation of ‘no smoking at home’	No	1			
	Yes	1	0.8–1.3	0.8	0.6–1.2
					
Smoking status	Current smokers	1			
	Current non-smokers	1.6	1.3–2	1.8	1.2–2.7

* 95% confidence interval

** odds ratio.

N=9,919 individuals from 656 PSUs of 6 strata.
